# Two decades of mucormycosis: molecular epidemiology and antifungal susceptibility trends in Türkiye

**DOI:** 10.3389/fcimb.2026.1771517

**Published:** 2026-03-06

**Authors:** Ayşen İkkan, Osman Merdan, Seçil Ak-Aksoy, Zeinep Chavouz Ametoglou, İmran Sağlık, Hazel Öztürk-Belik, Büşra Çalışır, Özlem Saraydaroğlu, Samet Kızıl, Beyhan Bülbül, Esra Kazak, Beyza Ener

**Affiliations:** 1 Department of Medical Microbiology, Faculty of Medicine, Bursa Uludağ University, Bursa, Türkiye; 2 Department of Infectious Diseases and Clinical Microbiology, Bursa Uludağ University, Bursa, Türkiye; 3 Department of Medical Pathology, Faculty of Medicine, Bursa Uludağ University, Bursa, Türkiye; 4 Department of Paediatric Infectious Diseases, Bursa Uludağ University, Bursa, Türkiye

**Keywords:** antifungal susceptibility, ITS sequencing, molecular epidemiology, Mucorales, mucormycosis, Türkiye

## Abstract

**Background:**

Mucormycosis is a life-threatening invasive fungal infection that mainly affects individuals with weakened immune systems. Despite its clinical importance, data from Türkiye on the epidemiology and antifungal susceptibility of fungi belonging to the order Mucorales are scarce. This study aimed to describe the clinical, epidemiological, and mycological features of mucormycosis over a twenty-year period at a tertiary care hospital.

**Methods:**

All cases of mucormycosis diagnosed between 2003 and 2022 at Bursa Uludağ University Hospital were retrospectively evaluated. Cases were classified as proven or probable according to international definitions. Molecular identification was based on internal transcribed spacer sequencing. Large subunit ribosomal DNA region sequencing and whole-genome sequencing were performed when necessary. Antifungal susceptibility testing for amphotericin B and posaconazole was performed according to the Clinical and Laboratory Standards Institute M38 guideline. Statistical analyses included chi-square, Fisher’s exact, Spearman’s correlation, and Wilcoxon signed-rank tests.

**Results:**

A total of 187 cases were identified, comprising 134 proven and 53 probable. Rhino-cerebral infection was the most frequent form, followed by pulmonary disease. Hematologic malignancy was the most common underlying condition, and mortality was 52.9 percent. Molecular identification was achieved for 99 isolates, with *Rhizopus arrhizus* predominant and *Rhizomucor pusillus* second. Posaconazole exhibited greater *in vitro* activity than amphotericin B (p < 0.001), while *Cunninghamella* species showed elevated amphotericin B minimum inhibitory concentrations.

**Conclusion:**

This single-center study provides the most comprehensive data on mucormycosis in Türkiye and highlights the importance of molecular surveillance to guide antifungal therapy and monitor regional variations.

## 1 Introduction

Mucormycosis is an invasive fungal disease with high morbidity and mortality rates. It generally occurs in immunocompromised patients, such as those with uncontrolled diabetes and hematologic malignancies, as well as transplant recipients. However, it can also occur in immunocompetent individuals following cutaneous inoculation through burns, insect bites, or trauma (Cornely et al., 2019). In addition, outbreaks associated with shared items, such as sheets, pillows, tongue depressors and bandages, have been documented in hospitals (Sundermann et al., 2019; Jordan et al., 2022). Furthermore, the COVID-19 pandemic has caused fatal co-infections in some patients (Sen et al., 2021).

Mucormycosis can present with several clinical presentations depending on the host’s immune status, the type of agent and the site of entry of the infection. Rhino–cerebral, pulmonary, cutaneous, gastrointestinal and disseminated mucormycosis are the most common presentations. Pulmonary mucormycosis occurs more frequently in patients with profound neutropenia and graft-versus-host disease, rhino-cerebral mucormycosis in patients with uncontrolled diabetes and cutaneous mucormycosis in immunocompetent individuals. Disseminated infection involves two or more non-contiguous sites (Cornely et al., 2019; Jeong et al., 2019).

Members of the order Mucorales, comprising about 250 species within 15 genera, are responsible for mucormycosis (Badali et al., 2021). However, the *Rhizopus*, *Mucor* and *Lichtheimia* genera contain the most disease-causing species, followed by *Rhizomucor*, *Cunninghamella*, *Apophysomyces* and *Saksenaea* species in humans (Jeong et al., 2019).

Culture is of great importance in the diagnosis of mucormycosis and allows species identification and antifungal susceptibility testing. In the latest published guideline, it is recommended at a high level of evidence (Cornely et al., 2019). Although routine species identification and antifungal susceptibility testing are not required for the Mucorales, it is now recommended epidemiologically due to the increasing incidence of mucormycosis and the occurrence of species with different susceptibilities in different geographical regions (Jeong et al., 2019; Badali et al., 2021). Unfortunately, identifying the Mucorales based on colony and microscopic morphology is very difficult and requires considerable experience. Because the rate of false identification is high, even in experienced centers, sequence analysis of the rDNA internal transcribed spacer (ITS) region is considered the gold standard for identification (Cornely et al., 2019; Chowdhary et al., 2014; Balajee et al., 2009).

There have been no epidemiological studies in Türkiye that have defined the order Mucorales at the species level and determined its antifungal susceptibility profiles. The present study focused on the epidemiological and clinical features of mucormycosis cases between 2003 and 2022 at our tertiary care hospital and aimed to identify Mucorales fungi at the species level and examine their antifungal susceptibility profiles.

## 2 Materials and methods

### 2.1 Study design and data collection

Bursa Uludağ University Hospital is an 800-bed tertiary-care teaching institution located in the South Marmara region of Türkiye, serving a catchment population of approximately 2–2.5 million people. As a regional referral center, the hospital provides specialized care for a large number of patients at increased risk for mucormycosis, including those admitted to medical and surgical intensive care units, patients with hematologic malignancies, recipients of allogeneic hematopoietic stem cell transplantation, solid organ transplant recipients, individuals with trauma, surgical wounds, or burn injuries, patients with autoimmune diseases receiving immunosuppressive therapy, and those with diabetes mellitus. Its mucormycosis cases between 2003 and 2022 were identified using pathology and microbiology laboratory information systems and infectious disease databases.

Proven cases were defined as those with positive culture and/or histopathological evidence obtained from biopsy material or sterile body fluids. Probable cases were classified according to the European Organization for Research and Treatment of Cancer/Mycoses Study Group Education and Research Consortium (EORTC/MSGERC) consensus criteria and required the simultaneous presence of host factors, abnormal clinical–radiological findings, and mycological evidence (Donnelly et al., 2020). Recognized host factors included hematologic malignancy, solid organ transplantation, prolonged corticosteroid exposure, and the use of T- or B-cell immunosuppressive agents. Mycological evidence for probable cases was based on recovery of Mucorales from clinically relevant non-sterile specimens from sites compatible with infection, and the clinical significance of culture results was determined according to predefined criteria, including repeated isolation from consecutive samples, growth on multiple culture plates from a single specimen, or positive direct microscopic examination of fresh clinical material. All other growth was considered contamination (Yang et al., 2015). Cases in which alternative pathogens were identified were excluded. All patients had received broad-spectrum antibacterial therapy without clinical improvement, and radiological findings were consistent with invasive fungal infection. Patients with clinical suspicion of disease but without mycological or histopathological confirmation were not included in the present study.

The cases’ demographic characteristics, underlying diseases, locations and infection types were obtained from the hospital information system. The Bursa Uludağ University Ethics Committee reviewed and approved the present study (2022, October 13).

### 2.2 Histopathological and microbiological examination

Histopathologic examination of clinical samples was performed by experienced pathologists. Proven infection was confirmed by the presence of pauci-septate, irregular, ribbon-like hyphae invading tissue in sections stained with hematoxylin and eosin (H&E), periodic acid–Schiff (PAS), or Grocott–Gomori’s methenamine silver (GMS) stain. For rapid preliminary diagnosis of mucormycosis, direct microscopic examination was performed using 10–30% potassium hydroxide (KOH) with lactophenol cotton blue, and hyphae consistent with members of the order Mucorales, like those observed in histopathological sections, were sought (Cornely et al., 2019).

During the study period, clinical samples were cultured on Sabouraud’s dextrose agar (SDA; Becton Dickinson [BD], Sparks, MD, USA), SDA containing chloramphenicol and gentamicin (BD), and brain heart infusion agar (BD) at 30 and 37 °C. All growths considered clinically significant were initially evaluated morphologically for features consistent with Mucorales and subsequently subjected to further identification procedures.

### 2.3 Molecular identification

In the present study, Mucorales strains were identified by molecular methods following amplification and sequencing of the ITS region. First, the stored isolates (one per patient) identified from patients during the study period were retrieved from the laboratory collections (-80°C) and incubated on SDA containing chloramphenicol and gentamicin (BD) at 35°C to ensure their viability and purity. DNA extraction from revived strains for molecular identification was performed both mechanically (glass bead disruption) and using a commercial kit. Briefly, portions of the mycelia were suspended in cetyltrimethylammonium bromide (Omega Bio-Tek, Norcross GA, ABD) and lysed using a bead beater instrument (Allsheng Bioprep 6; Allsheng Instruments Co., Ltd., China). Genomic DNA was then extracted using the E.Z.N.A.^®^ HP Fungal DNA Kit (Omega Bio-Tek) according to the manufacturer’s recommendations. The DNA was quantified using a UV-Vis spectrophotometer/NanoDrop (Beckman-Coulter, Brea Ca, ABD). ITS regions from the extracted DNA were amplified by polymerase chain reaction (PCR) using appropriate primers (ITS1 [5=-TCCGTAGGTGAACCTGCGG-3=] and ITS4 [5=-TCCTCCGCTTATTGATATGC-3=]) (Balajee et al., 2009; CLSI, 2018; Lu et al., 2013). Amplicons were purified (E.Z.N.A.^®^ Cycle Pure Kit [Omega Bio-Tek]) and sequenced by Sanger in the genetic analyzer (Beckman-Coulter CEQ8000, Brea Ca, ABD) using the Dye Terminator Cycle Sequencing Quick Start Kit (DTCS; Beckman Coulter). The sequences obtained were used to perform a Basic Local Alignment Search Tool (BLAST) search. A local BLAST database was built using ITS RefSeq sequences (https://ftp.ncbi.nlm.nih.gov/refseq/TargetedLoci/Fungi) (last modified: 2023 July 4) from the Fungal ITS RefSeq Targeted Loci Project (https://www.ncbi.nlm.nih.gov/bioproject/PRJNA177353) (Nilsson et al., 2015). Query sequences were aligned to the database using blastn (Version 2.12.0+) with megablast task (Zhang et al., 2000). All other general search options were left as defaults. Blastn species hits were considered significant, with an E value of 0.0 at 96%–100% identity and at least 95% coverage of the query sequence. Blast hits that did not meet these criteria were considered unidentified. Attempts were made to identify isolates that could not be identified through the ITS sequence by sequencing the D1/D2 regions of large-subunit (LSU) rDNA genes. The regions were amplified by PCR using primers (D1 [5′ GCA TAT CAA TAA GCG GAG GA]/D2 [5′ TTG GTC CGT GTT TCA AGA CG]) and sequenced by Sanger in the genetic analyzer (Beckman-Coulter CEQ8000) using a DTCS kit (Beckman Coulter) (CLSI, 2018; Hinrikson et al., 2005). Blast analysis was performed with the same criteria (http://www.ncbi.nlm.nih.gov/bioproject/PRJNA51803). When the sequencing of this region was also insufficient for identification, the whole genome was commercially sequenced.

### 2.4 Phylogenetic analyses

ITS sequences belonging to the same genera were aligned with MAFFT (Version 7.520) in auto mode, and multiple sequence alignment FASTA files were created (Katoh et al., 2005). IQ-TREE 2 (Version 2.2.0) was used to infer the maximum likelihood phylogenetic tree (Nguyen et al., 2015). The model was selected automatically by ModelFinder Plus, and branch supports were assessed with ultrafast bootstrap approximation (1,000 iterations) (Kalyaanamoorthy et al., 2017; Hoang et al., 2018).

### 2.5 *In vitro* antifungal susceptibility

Antifungal susceptibility testing for amphotericin B and posaconazole (Sigma-Aldrich, St. Louis, MO, USA) was performed according to the Clinical and Laboratory Standards Institute (CLSI) standard M38 (CLSI, 2017). Amphotericin B and posaconazole were diluted according to the recommendations in the reference method, and microdilution plates were prepared to obtain a final concentration of 0.03–16 μg/ml. Mucorales strains were grown on potato dextrose agar (BD), and after 48 h of incubation, the inoculum amount was adjusted spectrophotometrically at 530 nm (absorbance: 0.15–0.17). The spectrophotometrically adjusted suspension was diluted 1/50, and the final concentration was 0.4×10^4^–5×10^4^/ml. Microdilution plates were incubated at 35°C for 24 h, and the first well where growth was 100% inhibited was determined as the minimum inhibitory concentration (MIC). As no clinical breakpoints (CBP) have been determined for the CLSI method, susceptible/resistant interpretation was not done. For some species with epidemiological cutoff values (ECVs, 95% threshold), amphotericin B and posaconazole MICs were interpreted as wild type/non-wild type. Species-specific amphotericin B ECVs were taken as 1 µg/ml (*L. corymbifera* and *M. circinelloides*) to 2 µg/ml (*R. arrhizus* and *R. microsporus*), and posaconazole ECVs were taken as 1 µg/ml (*L. corymbifera*, *R. arrhizus* and *R. microsporus*) to 4 µg/ml (*M. circinelloides*) (Espinel-Ingroff et al., 2015; CLSI, 2022).

### 2.6 Data analysis

The patient characteristics and causative pathogens were summarized descriptively. The distributions of clinical forms across different underlying patient groups were compared using Pearson’s chi-squared or Fisher’s exact tests, as appropriate. *Post hoc* pairwise comparisons were then performed using Fisher’s exact test to determine significant intergroup differences. We calculated the incidence by dividing the total number of cases by the number of people confined in our hospital in each year of the study period. Spearman’s correlation analysis was applied to assess temporal trends in the occurrence of mucormycosis cases. The MIC ranges, MIC_50_, MIC_90_ and geometric mean (GM) MICs were calculated. The differences in GM MIC values between posaconazole and amphotericin B were determined using the non-parametric paired Wilcoxon signed-rank test (Alastruey-Izquierdo et al., 2009). Statistical analysis was performed using IBM SPSS Statistics 31.0 software (IBM Corporation, Armonk, NY, USA). A p value of ≤ 0.05 was considered statistically significant.

## 3 Results

In the present study, 187 cases of mucormycosis identified over a 19-year period were evaluated. Among them, 134 (71.7%) were classified as proven and 53 (28.3%) as probable. Direct microscopic examination and/or histopathological findings confirmed the disease in 125 (66.8%) cases, and Mucorales growth was observed in 76 (60.8%) of these cases. Sixty-two (33.2%) of the cases were identified by growth alone. The most common (65.2%) clinical form was proven rhino-cerebral mucormycosis, followed by pulmonary disease (29.9%) In addition, *Mucor circinelloides* was isolated from two separate blood culture sets obtained from a critically ill diabetic patient admitted to the intensive care unit. No sinonasal or pulmonary involvement was detected, and as no other microbial growth was identified, the finding was considered clinically significant and liposomal amphotericin B therapy was initiated. (**Table 1**).

**Table 1:** Presentation of mucormycosis cases.

Mucormycosis (number of patients)	Proven mucormycosis (n:134)			Probable mucormycosis (n:53)		
	Microscopic	Culture	Both	Microscopic	Culture	Both
Rhino–cerebral (122)^1^	46	18	58	–	–	–
Pulmonary (56)^2^	3	–	2	–	35	16
Cutaneous (8)^3^	–	6	–	–	2	–
Disseminate (1)^4^	–	1	–	–	–	–

^1^Most of the proven cases were confirmed by sino-rhinal biopsies. In four patients, the diagnosis was made through a brain biopsy.

^2^In four proven pulmonary cases, diagnosis was made through bronchoscopic biopsies; in one case, diagnosis was made through transthoracic biopsy. Probable cases were determined based on respiratory tract specimens.

^3^Proven infections were determined by growth in skin biopsy samples that developed after trauma (4) and surgery (2). In two probable cases, growth was observed in the swab specimens.

^4^A diabetic patient with blood culture growth of *Mucor circinelloides* and no other local foci.

The demographic characteristics and underlying conditions of the study population are summarized in **Table 2**. Most cases were adults (91.4%; p < 0.001). Although the number of male patients exceeded that of females, the difference was not statistically significant (p = 0.057). Hematological disorders represented the most prevalent (47.6%) underlying condition, followed by diabetes mellitus. Notably, diabetes coexisted in eight patients with hematologic malignancies, four patients with COVID-19, and two renal transplant recipients, resulting in a total of 47 patients (25.1%) with diabetes. Among solid organ malignancies, lung cancer was the most frequent (60%). The three patients without any identifiable underlying disease presented with proven cutaneous mucormycosis. The total mortality rate was 52.9% (n = 99).

**Table 2:** Demographic characteristics and underlying diseases.

Characteristic	Median (range) or No. (%)
Demographic characteristics (no = 187) • Age, median (range) years •Paediatric (0-18 years) patients (no = 16): •Adult (>18 years) patients (no = 171): • Sex, no. (%) •Males •Females	52 (3-86) 12 (3-17) 54 (19-86) 107 (57.2) 80 (42.8)
Underlying diseases, no. (%) • Haematological disease • ^1^AML: 49 • ^2^ALL: 25 • ^3^NHL: 5 • ^4^Other: 10 • Sole diabetes mellitus • ^5^Various chronic diseases • ^6^Solid organ malignancy • Renal transplant • COVID-19 • ^7^Other	89 (47.6) 33 (17.6) 27 (14.4) 20 (10.7) 9 (4.8) 6 (3.2) 3 (1.6)
Clinical forms, no (%) • Rhino–cerebral • Pulmonary • Cutaneous • Disseminate • Total	122 (65.2) 56 (29.9) 8 (4.3) 1 (0.5) 187

^1^AML, acute myeloid leukaemia; ^2^ALL, acute lymphoblastic leukaemia; ^3^NHL, non-Hodgkin lymphoma; ^4^Two cases each of bone marrow transplantation, myelodysplastic syndrome, multiple myeloma, aplastic anaemia and chronic lymphocytic leukaemia; ^5^Eighteen patients with chronic obstructive pulmonary disease, four patients with chronic renal failure and one patient each with systemic lupus erythematosus, Wegener disease, ulcerative colitis, pulmonary emboli and tuberculosis cavity; Cases classified as probable within the “various chronic diseases” group fulfilled EORTC/MSGERC host criteria (e.g., systemic corticosteroid exposure or other immunosuppressive conditions) together with compatible clinical–radiological findings and mycological evidence. Mucorales isolation was considered significant only when supported by repeated recovery from consecutive specimens, growth on multiple culture plates, or positive direct microscopy. Alternative infectious etiologies (bacterial, mycobacterial, or viral) were not identified, and all patients had received broad-spectrum antibacterial therapy without clinical improvement, thereby minimizing the likelihood of colonization or contamination. ^6^In addition to 12 lung cancer cases, there were two cases each of brain and bladder cancer and one case each of endometrial, bone, pancreas and stomach cancer; ^7^No underlying diseases.

Bold values denote the headings of each variable category.

**Figure 1:**
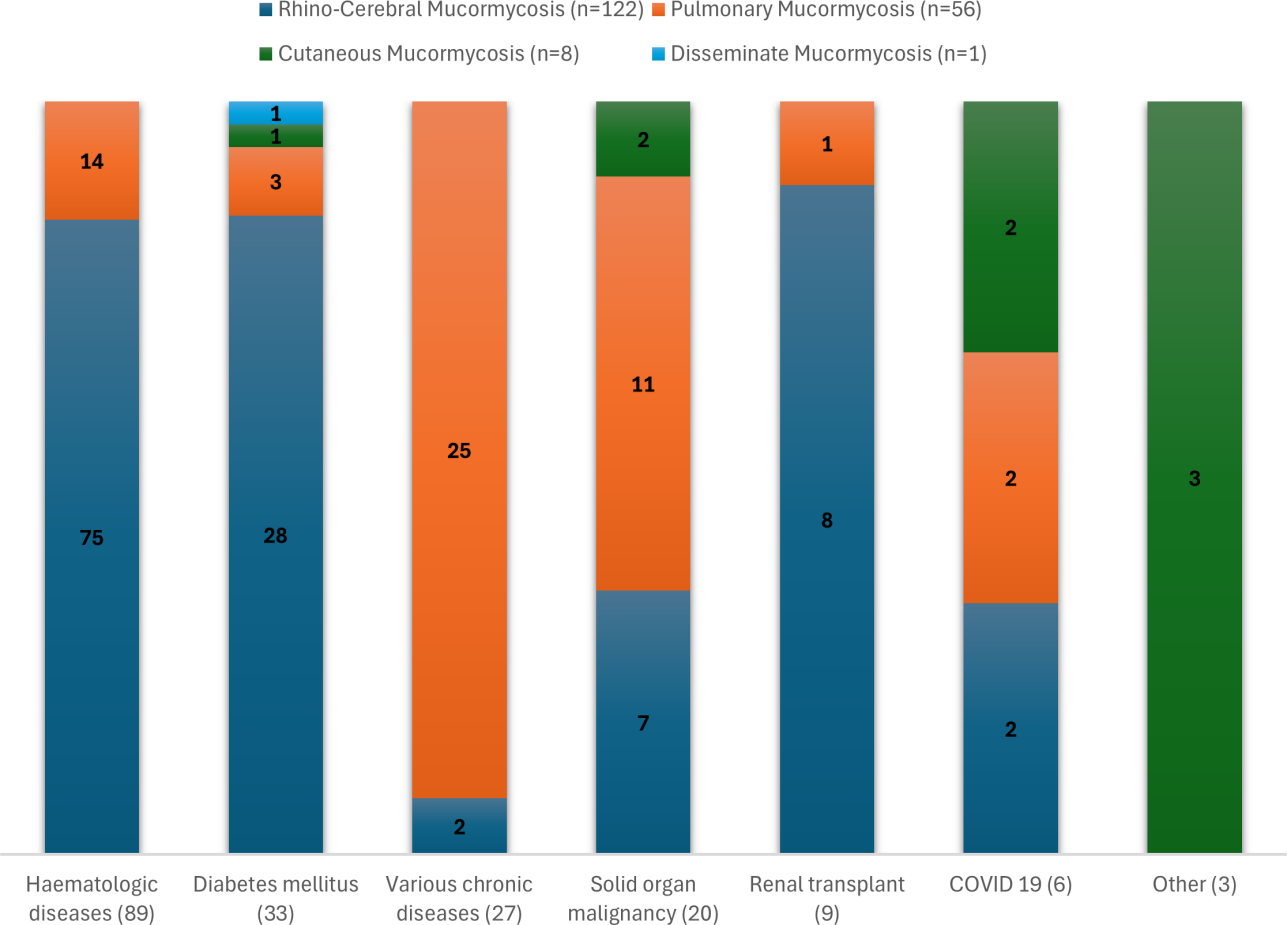
Distribution of mucormycosis clinical forms by underlying conditions. Stacked bar chart showing the within-group percentages of rhino-cerebral, pulmonary, cutaneous and disseminated presentations across patient groups (haematologic malignancies, diabetes mellitus, various chronic diseases, solid organ malignancies, renal transplantation, COVID-19 and other). Segment labels display the totals for each group.

**Figure 1** depicts the distribution of mucormycosis clinical manifestations in relation to underlying conditions. The rhino-cerebral form represented the most frequently encountered presentation, predominantly observed in patients with hematologic malignancies, diabetes mellitus or renal transplantation. However, no statistically significant differences were identified among these groups (Pearson’s chi-square test, χ² = 0.76, p = 0.68). In contrast, pulmonary mucormycosis was strongly associated with patients classified in the various chronic diseases group, in which most cases had underlying chronic obstructive pulmonary disorders (Fisher’s exact test, p < 0.001). Furthermore, pulmonary involvement was more frequent among patients with solid organ malignancies—most notably those with lung cancer—compared with patients with hematologic malignancies or diabetes mellitus (p ≤ 0.05).

**Figure 2:**
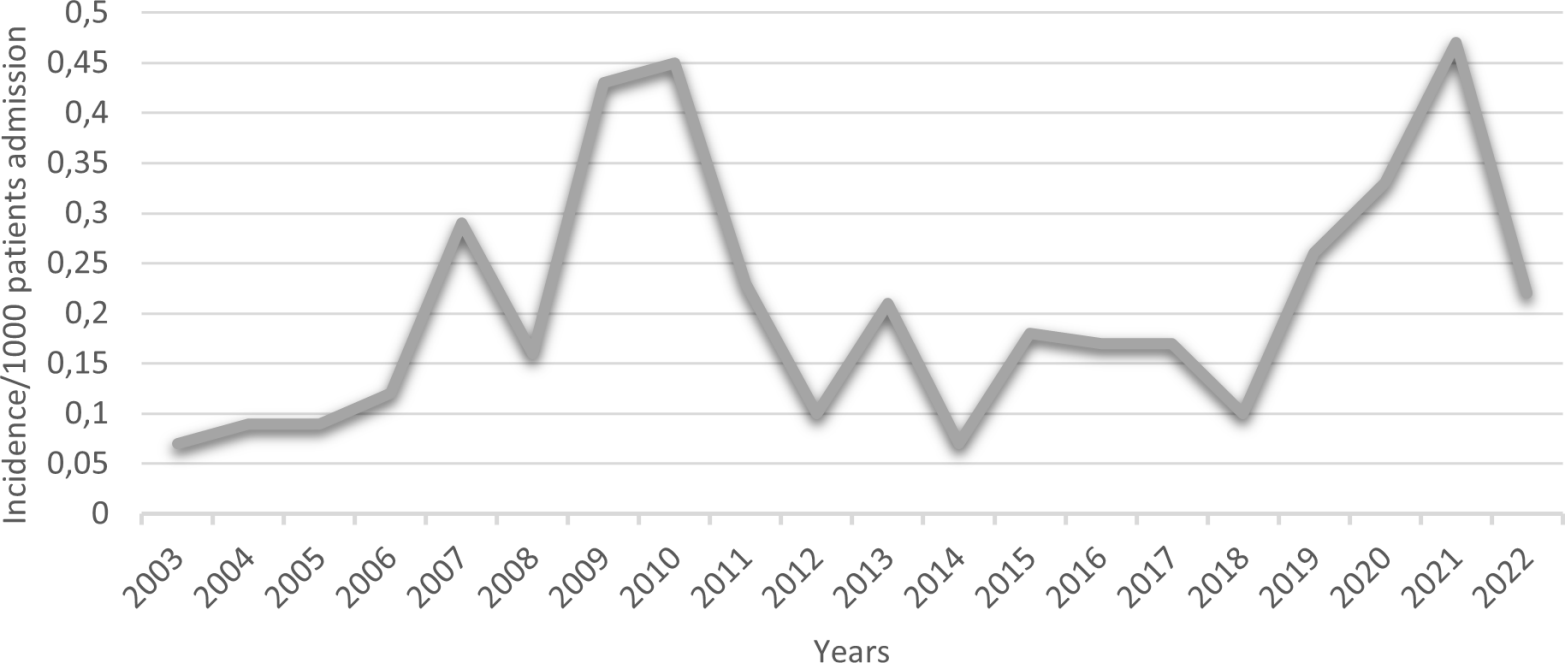
The Incidence of Mucormycosis (2003-2021).

The total number of patients admitted to our hospital during the study period was 939,068, and the incidence of mucormycosis per 1,000 patients was calculated to be 0.2. **Figure 2** illustrates the frequency of mucormycosis cases per 1,000 patients over the years. The highest rates (≥ 0.43) of mucormycosis were observed in 2009, 2010 and 2021, while the lowest rates (< 0.1) were recorded in 2003, 2004, 2005 and 2014. Although year-to-year fluctuations were noted, no consistent increasing trend or distinct surge during the COVID-19 pandemic period was observed. Spearman’s correlation analysis demonstrated a moderate positive correlation between years and incidence (r_s_ = 0.431), which did not reach statistical significance (p = 0.057).

Of the 187 mucormycosis cases included in the study, 49 were diagnosed solely by histopathology and therefore had no corresponding cultures available for recovery or further identification. Among the remaining 138 culture-positive isolates, 99 (71.7%) were successfully recovered and included in species-level identification analyses. The ITS region was sufficient for species-level identification in 88 (88.9%) of these isolates. *R. arrhizus* was the predominant species (66.6%), followed by *Rhizomucor pusillus* (14.6%). Six isolates with morphological features suggestive of *Cunninghamella* or *Syncephalastrum* were further characterized at the species level through sequencing of the LSU rDNA gene region. In addition, five isolates that were initially presumed to belong to the genus *Rhizopus* were conclusively resolved to the species level only through whole-genome sequencing (**Table 3**).

**Table 3:** Identified Mucorales species.

Mucorales species (n; %)	Number of isolates	Sequence region used	Data bases
*Rhizopus* species (71; 71.7) R. arrhizus R. microsporus R. delemar	66 4 1	ITS^1^ Whole genome Whole genome	RefSeq^2^ RefSeq RefSeq
*Rhizomucor* species (16; 16.2) R. pusillus R. miehei	14 2	ITS ITS	RefSeq RefSeq
*Lichtheimia* species (4; 4) L. ramosa L. corymbifera L. ornata	2 1 1	ITS ITS ITS	RefSeq RefSeq RefSeq
*Cunninghamella* species (4; 4) C. bertholletiae C. gigacellularis^4^ C. antarctica	2 1 1	28S 28S 28S	Public^3^ RefSeq RefSeq
*Mucor* species (2; 2) M. circinelloides	2	ITS	RefSeq
*Syncephalastrum* species (2; 2) S. monosporum	2	28S	RefSeq

^1^ITS, internal transcribed spacer; ^2^The ITS RefSeq Targeted Locus Project is a taxonomic initiative developed by the international mycological community to promote accurate and standardized nomenclature of fungal species. This effort was realized through the collaboration of key global fungal databases and institutions, including MycoBank, Index Fungorum, ISHAM and UNITE. ^3^Owing to the unavailability of *C. bertholletiae* in the RefSeq database, publicly accessible datasets were employed for the analyses. ^4^Upon comparison with publicly available sequence data, the isolate was classified as *Cunninghamella echinulata*.

**Figure 3:**
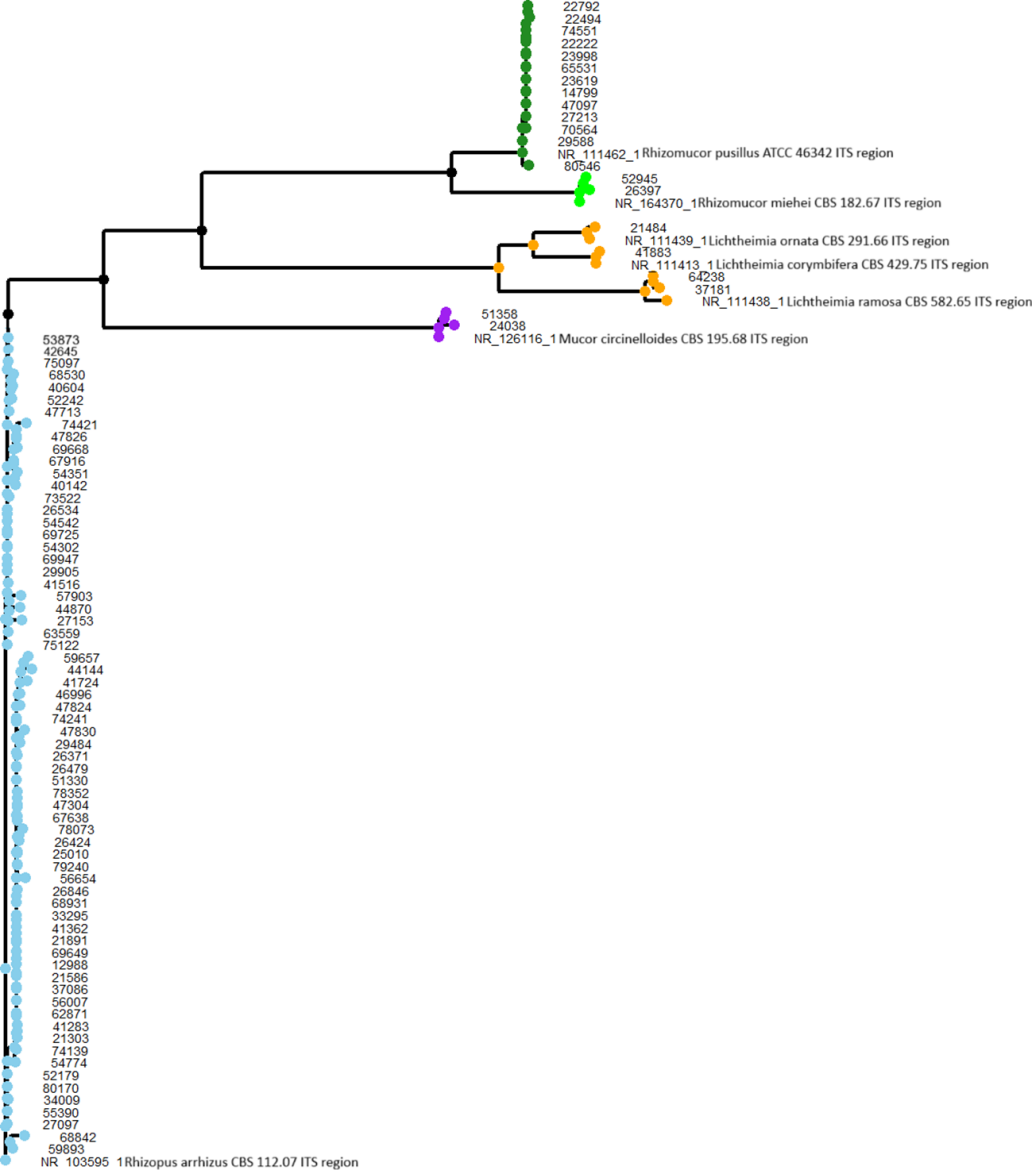
The phylogenetic analysis of Mucorales isolates. Genera are indicated by color-coded clades, allowing clear differentiation between *Rhizopus* (blue), *Lichtheimia* (orange), *Mucor* (purple), *Rhizomucor* (green).

**Figure 3** presents the results of the phylogenetic analysis, in which clear distinctions between all genera were observed. This unambiguous separation indicates that no errors occurred in the species identification process, confirming the accuracy of the results.

**Table 4** presents the *in vitro* antifungal susceptibility profiles of 99 clinical isolates. Posaconazole demonstrated superior overall activity compared to amphotericin B. The Wilcoxon signed-rank test revealed a statistically significant difference between the MIC values of the two agents (Z = –7.888, p < 0.001). Notably, elevated amphotericin B MICs were observed among *Cunninghamella* species, indicating a potential trend towards reduced susceptibility within this genus.

**Table 4:** *In vitro* antifungal susceptibility profile of the isolates.

Species	Antifungal	MIC (µg/ml)^1^					^2^NWT (%)
		MIC range	MIC_50_	MIC_90_	Mode	^3^GM	
*Rhizopus arrhizus* (66)	Amphotericin B	≤0.03 - 2	0.5	1	0.5	0.516	–
	Posaconazole	≤0.03 - 0.5	0.125	0.25	0.125	0.12	–
*Rhizopus microsporus* (4)	Amphotericin B	1 - 2					–
	Posaconazole	0.25-0.5					–
*Rhizopus delemar* (1)	Amphotericin B	1					
	Posaconazole	0.5					
*Rhizomucor pusillus* (14)	Amphotericin B	0.25 – 1	0.5	1	0.5	0.52	
	Posaconazole	0.06-0.5	0.25	0.25	0.25	0.19	
*Rhizomucor miehei* (2)	Amphotericin B	0.125-0.5					
	Posaconazole	0.03-0.06					
*Mucor circinelloides* (2)	Amphotericin B	1					–
	Posaconazole	0.125					–
*Cunninghamella bertholletiae* (2)	Amphotericin B	8					
	Posaconazole	0.25					
*Cunninghamella gigacellularis* (1)	Amphotericin B	8					
	Posaconazole	0.25					
*Cunninghamella antarctica* (1)	Amphotericin B	8					
	Posaconazole	0.25					
*Syncephalastrum monosporum* (2)	Amphotericin B	0.5					
	Posaconazole	0.06 – 0.25					
*Lichtheimia ramosa* (2)	Amphotericin B	1					
	Posaconazole	0.25-1					
*Lichtheimia cormbifera* (1)	Amphotericin B	0.5					–
	Posaconazole	0.125					–
*Lichtheimia ornata* (1)	Amphotericin B	1					
	Posaconazole	0.25					
Total (99)	Amphotericin B	≤0.03 - 8	0.5	2	0.5	0,68	
	Posaconazole	≤0.03 - 0.5	0.125	0.5	0.25	0,14	

^1^MIC, Minimum inhibitory concentration; ^2^NWT, Non wild type; ^3^GM: Geometric mean.

## 4 Discussion

This paper presents the results of a 20-year analysis of mucormycosis cases diagnosed at our center, in which Mucorales isolates were identified using molecular methods, and their antifungal susceptibility profiles were assessed. Despite being limited to a single institution, the present study represents the first comprehensive epidemiological investigation of mucormycosis in Türkiye, thereby providing valuable baseline data for future regional and international comparisons (Jeong et al., 2019; Troiano and Nante, 2022).

Consistent with previous reports, rhino-cerebral mucormycosis was identified as the predominant clinical presentation, with all such cases in our series meeting the criteria for proven infection (Roden et al., 2005; Alvarez et al., 2009; Kontoyiannis et al., 2014). Pulmonary mucormycosis was the second most common form; however, as also highlighted in earlier studies, challenges in obtaining histopathological confirmation from lung tissue often result in a higher proportion of cases being classified as probable rather than proven (Cornely et al., 2019; Guinea et al., 2017). Importantly, isolates without a clear clinical correlation—such as those demonstrating peripheral growth on culture plates or those recovered from a single specimen—were deliberately excluded. This stringent criterion was applied to minimize the risk of overestimation and to ensure that only clinically relevant infections were represented, an approach in line with recent consensus recommendations (Cornely et al., 2019; Yang et al., 2015; Guinea et al., 2017; Skiada et al., 2011; El Zein et al., 2018).

In our cohort, recovery of *M. circinelloides* from two separate blood culture sets was interpreted as true fungemia rather than contamination. Although bloodstream isolation of Mucorales is generally considered uncommon and culture positivity may occasionally raise concerns regarding contamination, true fungemia has been documented. Notably, a confirmed case of *M. circinelloides* fungemia was previously reported from Türkiye by Dizbay et al., in which the organism was recovered from blood cultures of a critically ill intensive care patient, supporting that such findings may represent genuine infection rather than contamination (Dizbay et al., 2009). Additional reports have similarly demonstrated repeated recovery of *M. circinelloides* from blood culture sets in clinically compatible hosts (Arroyo et al., 2016; Veltri et al., 2021). Furthermore, beyond classical angioinvasion, gastrointestinal colonization has been proposed as an alternative portal of entry for Mucorales; disruption of the gut microbiota and mucosal barrier integrity in critically ill patients may facilitate fungal invasion and subsequent translocation into the bloodstream, providing a plausible explanation for fungemia in the absence of an identifiable primary focus, as demonstrated in a recent study (Mueller et al., 2019).

Mucormycosis has long been recognized as one of the most common opportunistic infections in patients with diabetes mellitus. Diabetes remains the most important risk factor in countries where adequate glycemic control is not consistently achieved. However, in settings where diabetes is well controlled, malignancies—particularly hematologic malignancies—constitute the predominant underlying condition (Cornely et al., 2019; Jeong et al., 2019; Skiada et al., 2011). Because our hospital is the largest center for cancer patient follow-up in the southern Marmara region of Türkiye, hematologic malignancies constitute the largest patient group in this cohort. Although rhino-cerebral mucormycosis is most frequently observed in patients with diabetes mellitus, in the present study, its occurrence was similarly high among patients with hematologic malignancies. At our center, the routine practice of obtaining specimens with the slightest clinical suspicion likely reduced the risk of missed diagnoses. We consider this practice, combined with the predominance of hematologic malignancies within our study population, to have contributed to the relatively high frequency of rhino-cerebral mucormycosis observed in the diabetic, renal transplant and hematologic malignancy groups (Roden et al., 2005; Skiada et al., 2011; El Zein et al., 2018). Although pulmonary involvement is most commonly associated with hematologic malignancies, pulmonary mucormycosis was primarily associated with patients who had chronic pulmonary disorders, such as chronic obstructive pulmonary disease and lung cancer, in the present study. These observations are consistent with those in prior studies demonstrating that pre-existing structural lung disease, impaired mucociliary clearance and reduced pulmonary immune defenses facilitate the establishment of pulmonary mucormycosis (Jeong et al., 2019; Roden et al., 2005).

Determining the incidence of mucormycosis is difficult because of the lack of standardization in diagnostic criteria and low awareness of the disease. Thus, epidemiological studies of mucormycosis are inadequate due to the limited number of confirmed cases. The incidence may be underestimated due to the difficulty of diagnosis or overestimated due to false culture positivity (colonization or contamination). Although mucormycosis occurs worldwide, its incidence is particularly high in India and Iran (Chowdhary et al., 2014; Dolatabadi et al., 2018). In the present study, the incidence was found to be slightly higher than that reported in previous studies. The likelihood of overestimation is reduced by the fact that all rhino-cerebral cases, 75% of cutaneous cases, and 71.1% of all cases in our cohort were classified as proven. Furthermore, approximately two-thirds (63.1%) of our study group consisted of immunocompromised patients, such as those with hematologic malignancy, solid organ tumors and renal transplantation, which is consistent with the literature, suggesting a higher incidence in this patient group (Cornely et al., 2019).

In the present study, molecular identification was performed on 99 culture-positive isolates that were successfully recovered, while a proportion of culture-positive isolates could not be revived from long-term storage, potentially influencing the observed species distribution. Comprehensive epidemiological investigations have consistently demonstrated that *Rhizopus* species represent the predominant etiological agents of mucormycosis, accounting for approximately 40%–79% of all isolates (Jeong et al., 2019; Badali et al., 2021; Roden et al., 2005; Bonifaz et al., 2021). In line with these findings, *Rhizopus* species were also the most frequently identified fungi (75%) among the 88 isolates characterized by ITS sequencing in the present study. Previous studies have generally reported *Mucor* and *Lichtheimia* species as the second and third most common genera, respectively (Badali et al., 2021; Bonifaz et al., 2021). However, in contrast to these reports, our study identified *R. pusillus* as the second most prevalent species, constituting a substantial proportion (14.1%) of the isolates. Geographical variation in the distribution of Mucorales species has been well documented. For instance, *Lichtheimia* species are reported to be more common in Europe, while *Apophysomyces variabilis* represents the second most frequently isolated species in India, accounting for nearly 60% of *Apophysomyces*-associated mucormycosis cases (Skiada et al., 2011; Chakrabarti et al., 2003). *Mucor irregularis*, on the other hand, has predominantly been recovered from cases in China and India (Hemashettar et al., 2011). These observations underscore the strong influence of geography on species distribution. As no other large-scale epidemiological investigations of mucormycosis have been conducted in Türkiye, it remains uncertain whether the relatively high prevalence of *R. pusillus* observed in the present study reflects a regional characteristic or an isolated finding. Further comprehensive, multicenter studies are warranted to elucidate this issue. In addition, previous studies have demonstrated the frequent detection of *R. pusillus* spores in hospital environments, including bed linens, air, showers and ventilation systems, implying the potential for nosocomial transmission or outbreaks (Walther et al., 2019). In the present study, however, no clustering of mucormycosis cases was observed; therefore, a hospital-acquired infection was not considered likely. Moreover, because environmental sampling was not performed, the potential role of environmental contamination within our institution could not be assessed.

In the present study, 11 isolates could not be identified using the ITS region. This finding is consistent with previous reports suggesting that the ITS region alone may be insufficient for the accurate identification of Mucorales species. In such cases, the use of the D1–D2 region of the LSU as an additional molecular marker has been recommended (Kurahara et al., 2024; Mousavi et al., 2019; Raju et al., 2020). In our dataset, the application of the D1–D2 region of LSU sequencing enabled the successful identification of six isolates, four of which were classified as *Cunninghamella* and two as *Syncephalastrum*. Moreover, the five isolates that remained unresolved after ITS and LSU analyses were subjected to whole-genome sequencing, which allowed for definitive species-level identification (Cornely et al., 2019).

In the present study, the *in vitro* antifungal susceptibilities of 99 Mucorales isolates were determined using the CLSI M38 reference method (CLSI, 2017). Because no definitive *in vitro*–*in vivo* correlation has yet been established for the order Mucorales, the clinical interpretation of MIC data remains challenging. Although ECVs have been proposed for certain species, CBPs are still lacking for both the CLSI and EUCAST methods (Espinel-Ingroff et al., 2015). In our series, elevated amphotericin B MICs were observed among *Cunninghamella* isolates, consistent with previous evidence indicating reduced susceptibility or intrinsic resistance within this genus (Almyroudis et al., 2007). The high amphotericin B MIC values identified in *Cunninghamella* species in the present study further support emerging evidence of intergeneric variability in polyene susceptibility. Conversely, posaconazole consistently demonstrated lower MICs across isolates, reinforcing its role as an effective therapeutic option. Recent multicenter surveillance studies have also shown that antifungal susceptibility patterns can vary across regions and time periods, with some cohorts reporting higher activity of amphotericin B, while others confirming the superior potency of posaconazole (Raeisabadi et al., 2025; Badali et al., 2025; Bernauer et al., 2025). Collectively, these findings emphasize that antifungal treatment decisions should be guided not solely by historical paradigms but also by contemporaneous local and regional susceptibility data to optimize therapeutic outcomes in mucormycosis.

In conclusion, although the present study was conducted at a single center, it is the most extensive epidemiological study of mucormycosis in Türkiye to date, integrating both clinical data and molecular species identification. Rhino-cerebral mucormycosis emerged as the predominant clinical manifestation, while hematologic malignancy was the most common underlying predisposing condition. The comparatively high incidence observed in this cohort likely reflects the large proportion of immunocompromised patients included. In alignment with international data, *R. arrhizus* was the leading etiologic agent; however, *R. pusillus* ranked second, in contrast to the findings of most previously published studies. These observations suggest possible geographic or environmental differences in the distribution of Mucorales species and highlight the necessity of ongoing regional surveillance. Although ITS region sequencing provided adequate resolution for most isolates, it was insufficient for precise differentiation of species beyond *R. arrhizus*, *Cunninghamella* and *Syncephalastrum*, highlighting the need for complementary loci or whole-genome sequencing approaches in future investigations.

## Data Availability

The sequencing data are publicly available in the NCBI Sequence Read Archive (SRA) under accession number PRJNA1391998 (https://www.ncbi.nlm.nih.gov/sra/PRJNA1391998).
